# Sleep in Hospitalized Patients

**DOI:** 10.3390/clockssleep1010014

**Published:** 2019-02-25

**Authors:** Anne Marie Morse, Evin Bender

**Affiliations:** 1 Geisinger Commonwealth School of Medicine, Department of Child Neurology and Sleep Medicine Geisinger Medical Center, Janet Weis Children’s Hospital, 100 N. Academy Ave, Danville, PA 17820, USA; 2 Department of Neurology, Geisinger Medical Center, 100 N. Academy Ave, Danville, PA 17820, USA

**Keywords:** hospitalized patients, sleep wake dysfunction, sleep disorders, circadian rhythm, sleep apnea

## Abstract

Hospitalized patients frequently have disordered and poor-quality sleep due to a variety of both intrinsic and extrinsic factors. These include frequent nighttime intrusions, insomnia related to pain and unfamiliar environments, dark conditions during the day with loss of natural light, and disruption of the natural sleep cycle due to illness. Sleep wake disturbances can result in a deleterious consequence on physical, emotional, and cognitive status, which may impact patient satisfaction, clinical recovery, and hospital length of stay. Despite this, clinicians frequently fail to document sleep disturbances and are generally unaware of the best practices to improve sleep quality in the hospital. A PubMed search was conducted using the terms: (“sleep and hospitalized patients”) and (“sleep and hospitalization”) to review the published data on the topic of sleep in hospitalized medical patients. The search was limited to English-language articles published between 2000 and 2018. Subsequent PubMed searches were performed to clarify the data described in the initial search, including the terms “hospital sleep protocols,” “hospitalized patients sleep documentation,” and “hospitalized patients sleep quality”. The purpose of this review is to discuss sleep disturbances in hospitalized patients with a focus on causes of sleep disturbance, the effect of poor-quality sleep, high risk populations, considerations for surveillance and prevention, and pharmacologic and non-pharmacologic options for treatment.

## 1. Introduction 

Sleep is a homeostatic process that contributes to the maintenance of health, cognition, and mood stability [1,2]. Normal sleep in an adult is generally about 7–9 h per night and consists of 4–6, 90–120 min cycles between non-rapid eye movement (REM) and REM sleep [3]. Sleep wake dysfunction (SWD) refers to disruption of the normal sleep wake relationship that can lead to the development of a transient or chronic sleep wake disorder (SD). This can be a result of a reduced number of hours of sleep, poor quality sleep, or inappropriate timing or stabilization between wake and sleep. When present, SWD can contribute to the development and progression of co-morbid disease. 

Approximately 10% of adults have a chronic SD that results in clinically significant symptoms [4], with up to 50% of adults experiencing transient episodes of sleep wake dysfunction. Similarly, in pediatrics, 20%–30% of children are affected by a sleep disorder [5] and up to 80% of children with chronic conditions have a co-morbid sleep disorder [6,7,8]. The risk of development of a chronic sleep wake disorder increases with age [9]. Patients with chronic medical disease are at a higher risk for experiencing co-morbid sleep wake disorders [10]. In fact, acute hospitalization for medical illness is a high-risk period for the development or exacerbation of SWD, which can result in deleterious consequences [9,11,12,13,14,15]. 

Hospitalized patients frequently report sleep quality while an inpatient to be similar to the report of sleep quality of individuals who suffer from chronic SD [2]. For instance, insufficient sleep, with an average of 5 h of sleep per night, with multiple night time awakenings, has been found in adult patients in a neurologic ward [1]. There is a suggestion that the higher the acuity of illness, the greater the likelihood of developing sleep dysfunction. For instance, patients in the ICU are more likely to have severely disrupted sleep with a disturbed to loss of the circadian pattern, decreased total sleep time, abnormal sleep architecture with increased stages 1 and 2, and sleep with reduced to absent N3 sleep. 

Hospitalization in pediatric patients frequently results in sleep disruption for both the child and the parent [16,17,18,19,20,21]. For example, pediatric patients in an intensive care unit can experience up to a 54 percent reduction in [22] sleep hours, while children in the general pediatric unit have been reported to experience a 20%–25% sleep loss [23]. In younger hospitalized children, sleep disruption has been related to later bedtimes, later wake times, sleep fragmentation, and a reduced total sleep time [18]. Hospitalized adolescents also had later wake times and sleep fragmentation, but frequently reported a longer total sleep time [18]. A closer examination of the causes of poor sleep, identification of high risk populations, and the consequence of impaired sleep complemented with a review of surveillance techniques and management strategies for SWD may assist in improving early recognition and treatment of sleep disorders in hospitalized patients. 

## 2. Sleep Wake Dysfunction in Hospitalized Patients

The most commonly acquired SD in hospitalized patients are insomnia and circadian rhythm disorders [24,25,26,27]. However, sleep disordered breathing (SDB), restless leg syndrome (RLS), and hypersomnia disorders can also be present and may be pre-existing or acquired, depending on medical co-morbidity [11,28]. If there is pre-existing SD, this may become exacerbated by hospitalization and may predispose to the development of additional SWD. 

There are both intrinsic and extrinsic factors that contribute to the development of SWD during hospitalization [3,29,30,31]. Intrinsic factors vary by patient, but can include primary medical illness, delirium, psychiatric co-morbidity (anxiety, depression, post-traumatic stress), pre-existing SD, and physical pain or discomfort [1,2,32,33]. Pain and discomfort are generally the most frequently cited causes of SWD during hospitalization [1]. 

Extrinsic factors that contribute to the development of SWD can be environmental noise, bright lights, and irregular exposure to lighting; an unfamiliar environment with the loss of a normal bedtime routine; and repetitive clinical interventions, such as testing, clinical examinations, and vitals, as well as medication administration [18,34]. Environmental noise can be due to equipment/alarms, medical staff, or hospital roommates. Roommates are commonly reported as the most disturbing factor for sleep [1]. A study evaluating acceptable environmental noise levels found that daytime noise of 59 decibel A (dBA) and nighttime noise of 50.5 dBA did not result in SWD [35]. However, median noise during the day was found to be 63 dBA and 61 dBA at night [35]. Noise levels in the ICU have been documented at an average peak of 150–200 dB, with evening peaks >80 dB between 12 AM and 6 AM, contributing to about 30% of patient awakenings [36].

Bright lighting and lighting irregularities may represent a more easily modifiable extrinsic factor for hospitalized patients. The strongest environmental cue for human circadian rhythm is the light dark cycle [37]. Therefore, both irregular light exposure and bright light exposure during inappropriate circadian hours can result in circadian sleep dysfunction. Circadian misalignment has been demonstrated to be deleterious in multiple disease processes, resulting in worsened disease severity, an impaired treatment response, and even decreased survivorship [14]. 

## 3. Effects of SWD in Hospitalized Patients

**Figure 1:**
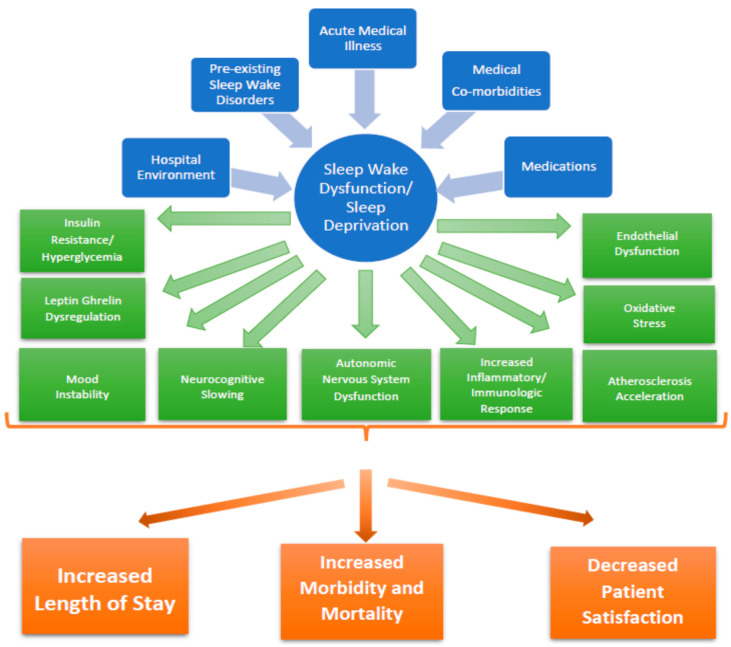
Consequences of Sleep Wake Dysfunction and Sleep Deprivation [1,2,9,11,12,13,14,15].

Sleep wake disorders can result in a deleterious consequence on physical, emotional, and cognitive status, which may contribute to impaired recovery, prolonged length of stay, reduced subjective wellness, and poor patient perception of hospitalized care [1,2,33,38]. SWD and sleep deprivation have numerous deleterious effects (Figure 1), including autonomic dysfunction, impairment of the hypothalamic-pituitary-adrenal axis, and immunologic dysregulation [11]. Among hospitalized patients, reduced sleep duration and impaired sleep efficiency were independently associated with a greater likelihood for impaired fasting glucose and hyperglycemia [15]. 

Poor quality sleep increases the likelihood of the development of delirium, anxiety, and mood disorders [39,40,41]. Delirium and sleep deprivation have shared characteristics, including impaired attention, fluctuating mental status, disorganized thinking, and an altered level of consciousness [40]. Delirium occurs in up to 50% of hospitalized elderly patients, with strong associations in those greater than 80, and is more common in men [38,40]. Delirium increases length of stay, likelihood for institutionalization post discharge sevenfold, and risk for six-month mortality when compared to those without delirium [38]. 

The presence of pre-existing or newly developed SWD can significantly impact clinical outcomes. The most well-studied co-morbid sleep disorder in hospitalized patients has probably been obstructive sleep apnea (OSA) [28,42,43,44]. Surgical patients with OSA are more likely to experience postoperative hypoxemia and require ICU transfers with longer hospital stays [28]. Similarly, pregnant women with co-morbid OSA have an increased post-partum risk for maternal morbidity, cardiovascular morbidity, and in-hospital death [42]. 

Circadian rhythm disturbances are gaining attention as a cause of SWD that negatively contributes to outcome measures in hospitalized patients [14,26,27]. The circadian rhythm does not only influence sleep wake patterns, but also dictates physiological and cellular functions that allow for adaption to cyclic environmental changes [45]. Evidence suggests that the relationship between circadian function and disease is quite complex and multidirectional [14,26,27,37,45,46,47,48]. There is a loss of normal diurnal variation in melatonin secretion in critically ill patients [48], likely contributing to circadian misalignment. Patients hospitalized in the ICU have a loss of the normal rhythmic 24-h physiologic profiles, including blood pressure, heart rate, body temperature, spontaneous motor activity, and the levels of melatonin and cortisol [49]. In addition, there is a suggestion that there is a circadian effect on survival, which has been suggested to be related to the circadian variability of immune responses [47].

## 4. High Risk Populations for SWD

There are multiple factors that may influence the development of SWD during hospitalization (Table 1). Duration of slow wave sleep (SWS), total REM sleep, and arousal thresholds decrease with age, culminating in reduced total sleep times with increased sleep fragmentation in individuals greater than 60 years old [50,51]. In addition to the natural sleep changes that occur with age, advancing age is generally related to the development of comorbid medical conditions. It is not uncommon for elderly patients to describe sleep complaints due to the presence or complications of these comorbidities [52]. 

Comorbidities commonly associated with sleep disordered breathing and obstructive sleep apnea (OSA) include heart failure, coronary artery disease (CAD), and stroke, among others [59,60,61,62,63,64,65]. It is critical to identify OSA and sleep disordered breathing in these particular at risk populations because untreated OSA is an independent risk factor for initial and recurrent stroke, promotes CAD, and nearly triples the risk of mortality in patients with CAD [63,64,65,66]. Diabetes is another condition associated with sleep changes, and it has been shown to correlate with lighter sleep with an increased percent of time spent in stage 1 and 2 of sleep [50]. COPD has also been shown to correlate with a poor quality of sleep due to increased sleep latency, a decreased total sleep time, and increased arousal [67]. Parkinson’s disease correlates with sleep fragmentation and REM behavior disorder (RBD), which can be particularly challenging to address in a hospital setting [68]. Additionally, many conditions are associated with insomnia, the most extensively studied of which include cancer, untreated or poorly treated nocturnal pain, psychiatric conditions such as depression and anxiety, heart disease, and neurologic disease [34,52,58,69,70]. 

Women have a slight predominance in the occurrence of insomnia, as well as periodic limb movement disorder (PLMD) and restless leg syndrome (RLS) [56,57]. They are also statistically more likely to present to medical attention for sleep complaints and are therefore more likely to be prescribed hypnotic medications [57]. Men, on the other hand, have a higher occurrence of OSA than women, and tend to have lighter sleep with a greater time spent in stage 1 and stage 2 sleep compared to women. They also have a slight predominance for narcolepsy, and older men (age >50) have a slight predominance for REM behavior disorder compared to women [50,57]. Sleep changes associated with older age include lighter sleep and a decreased sleep efficiency with a greater time spent in stage 1 and 2 sleep [50]. Older age was also associated with a greater occurrence of nocturnal myoclonus, RLS, and sleep disordered breathing [51].

In addition to age, sex, and co-morbid conditions, many medications also are associated with SWD. Stimulant medications are associated with insomnia and circadian rhythm disorders [69]. Benzodiazepines and opioids decrease the percent of time spent in later stages of sleep, and thereby reduce sleep efficiency [71,72]. Antidepressants also reduce the time spent in REM sleep and increase REM onset latency; however, this typically normalizes with long-term treatment. An exception to this includes MAOIs, which can cause REM sleep to be absent for months [73].

At first glance, it appears that all those hospitalized are at an equally high risk for sleep wake dysfunction. However, it is important to clarify considerations when approaching sleep in hospitalized patients. There is variability across age, gender, co-morbidity, and severity of illness. For instance, elderly men are at a higher risk for sleep disordered breathing. This risk may be compounded in the setting of hospitalization for myocardial infarction or stroke requiring ICU admission. This is illustrated in a study that evaluated 180 patients with acute myocardial infarction, of which 83 percent were male. It was found that there was an increased prevalence of previously undiagnosed OSA patients, which resulted in higher pulmonary artery systolic pressure [74]. On the other hand, adolescent females are at a higher risk for sleep initiation difficulties with associated mood dysfunction. This is illustrated in a 15-year follow-up of 9000 adolescents which identified that adolescents who were hospitalized for self-harm were primarily female (73%) and a history of sleep initiation difficulties with anxiety/depressive symptoms was strongly associated with SH hospitalization [75]. These differences should be considered when choosing surveillance techniques and developing inpatient sleep protocols.

## 5. Physician Surveillance of SWD

Despite the high prevalence of chronic sleep disorders in the general population and the knowledge that acute hospitalization is a high risk for the development or exacerbation of an SWD, physicians frequently fail to address and document SWD in the hospital. One study performed on the general medical ward of a Veteran’s Affairs hospital found that although 23% of patients admitted had a significant sleep symptom, there was no medical record during their admission documenting sleep symptoms [76]. Another study performed on a general medical floor at a non-profit academic hospital found that sleep histories were documented on only 9% of hospital charts, despite 68% of patients having conditions associated with obstructive sleep apnea (hypertension, obesity, stroke, and prior myocardial infarction) [77]. 

Physician awareness of available screening tools may influence likelihood to consider sleep. Standardized screening (Table 2) should be performed at admission to improve the recognition of those with pre-existing sleep disorders and may help capture individuals who are at a higher risk for the development of SWD during hospitalization. Re-evaluation during hospitalization may allow identification of those with secondary development of SWD and provide opportunity for intervention. The screening tools suggested have not been validated specifically for hospitalized patients. Despite this, utilization may still offer value in identifying high risk sleep characteristics in hospitalized patients. The authors utilize the SDS-25 CL as a screening tool, owing to its comprehensive review, but brevity to complete (generally less than 3 min). However, some may prefer more specific evaluations, such as the STOP-BANG or Berlin Questionnaire, which specifically screen for sleep apnea, due to a perception of greater ease for implementation or an understanding of the relationship of OSA to morbidity and mortality relative to other sleep dysfunction. 

Proactive methods to prevent the development of SWD in hospitalized patients may include the implementation of a sleep wake protocol that considers patient risk factors, hospital environment, and timing of interventions coupled with the reinforcement of regular sleep scheduling, sleep hygiene, and pharmacologic interventions as needed [1,37]. Utilization of a standard sleep wake protocol (Table 3) in our intensive care unit has resulted in a reduction in the development of delirium in our critical care patients. Patient responses to sleep disorder screening tools may better inform the development and implementation of sleep wake protocols. Research evaluating the efficacy and impact of these preventative strategies, however, is limited. 

## 6. Management of SWD in Hospitalized Patients 

### 6.1. Non-Pharmacologic Interventions 

Nonpharmacologic approaches to the treatment of sleep disturbances should be considered and implemented first for sleep difficulties in hospitalized patients. Non-pharmacologic interventions include strategic lighting, noise reduction techniques, and sleep rounds with sleep protocols featuring various implementations to aid with relaxation. A study on daytime light exposure and sleep found that exposure to at least 3000 lx between the hours of 10:00 and 15:00 was likely to improve the quality and duration of sleep in elderly patients [84]. Supportive care directed at improved relaxation, such as the use of warm blankets, warm milk, a white noise machine, hypoallergenic lotion, or a room spritzer, has been shown to improve perceptions of noise levels and reduce sleep latency in hospitalized patients [1]. The use of a three part sleep protocol, not only resulted in improved patient’s self-reported sleep quality, but also significantly reduced the use of sedative hypnotic drugs from 54% to 31% [32].

While elderly and acutely ill patients are at risk for SWD while hospitalized, they are also more prone to serious adverse effects of sedative hypnotic drugs, including falls, delirium, and respiratory depression [33]. Therefore, nonpharmacologic approaches for sleep may be useful as a prophylaxis against sleep and circadian disruption, in addition to therapeutic intervention. Sleep protocols that enhance circadian maintenance and promote a dark quiet sleep environment with reduced interruption may reduce the risk for the development of SWD. However, at this time, there is insufficient evidence to support the idea that non-pharmacologic interventions improve sleep quality or quantity of general inpatients [85]. It is important to consider that the lack of evidence supporting nonpharmacologic success may be related to the significant variability in the types of interventions applied, outcome measures, follow-up time, and differences in patient populations [85].

### 6.2. Pharmacologic Interventions

Sedative-hypnotic drug (SHD) use is common, both in the general population and among hospitalized patients. Approximately 3% to 6% of hospitalized children are treated pharmacologically with sleep medications [19]. In adults, approximately 35% of patients are prescribed a home hypnotic for sleep [2], and 45%–70% of patients are prescribed a hypnotic during their hospitalization [33]. Additionally, approximately 30% of patients are prescribed a new hypnotic in the hospital without any record of pre-admission use [2]. When SHDs are used, pharmacokinetics, an adverse effect profile, and cost should be considered [86]. The majority of SHDs used include benzodiazepines or zopiclone, but it is not uncommon for antidepressants, antipsychotics, antiemetics, and narcotics to be used for their sedating properties [2]. Melatonin is frequently used, with varied success [87,88]. In ICU patients, who are at a high risk for circadian dysregulation, there is insufficient evidence to determine if melatonin would improve the quality and quantity of sleep [88]. It has been suggested that intermediate-acting benzodiazepines, such as lorazepam or temazepam, would be good first-line agents, with zaleplon and zolpidem being second-line due to cost [86]. Alternatively, trazodone may be considered for patients unable to take benzodiazepines [86]. Although the intention of sedative hypnotics is to improve sleep, use of these medications has not consistently been proven to correlate with improved sleep quality, but instead, has been associated with adverse cognitive outcomes and falls [2]. 

Depending on the clinical situation, an SHD may not be indicated and instead, a wake promoting agent or stimulant may be recommended. For instance, a young man with prolonged hospitalization suffered nightly sleep difficulties. He had been prescribed melatonin, lorazepam, and zolpidem, without improvement. Sleep consultation revealed an irregular sleep wake pattern (i.e., sleep fragmented across 24 h), which improved with a combination of behavioral modification (daytime out of bed, sleep scheduling, stimulus control) plus a wake promoting agent. Wake promoting agents and stimulant therapy may be helpful in the setting of hypersomnia disorders and in some cases of circadian dysfunction [89].

## 7. Discussion 

An improved understanding of the various factors that can influence sleep during hospitalization allows providers to better evaluate and treat sleep problems more systematically, but also creates an opportunity for systems to adapt preventative and surveillance practices. Sleep wake dysfunction is a multifactorial problem in hospitalized patients, resulting from a combination of the hospital environment, psychological stress, the patient’s medical illness, and treatments. Patients can be stratified by these features that may increase the likelihood of developing sleep difficulties during hospitalization (Table 1). 

Implementing standardized screening for SD at admission and possibly during admission may help identify those with pre-existing sleep disorders and those who may have developed SWD during hospitalization. Treatment of SWD should be provided with both pharmacologic and non-pharmacologic options considered. Non-pharmacologic options are typically safer and have been shown to be beneficial. Proactive methods to prevent the development of SWD in hospitalized patients may be considered. Further research is needed to evaluate the efficacy in preventing SWD, as well as impact on length of stay, patient’s perception, morbidity, and mortality. 

## 8. Conclusions

Sleep is a frequently neglected component of a patient’s history during hospitalization that can influence length of stay, morbidity and mortality and patient satisfaction. Both pre-existing and newly acquired sleep wake disorders can have deleterious consequence, if left unrecognized and untreated. Standardized inpatient sleep wake protocols should be tailored by institutions to improve acquisition of histories of pre-existing SWD, promote an environment that reinforces normal sleep wake cycles, and provide recommendations on management options for SWD while inpatient. 

## Figures and Tables

**Table 1:** Risk Factors for SWD in Hospitalized Patients [9,10,11,12,14,15,26,27,29,50,53,54,55,56,57,58].

Risk Factor	High Risk Groups	Clinical Signs and Symptoms
**Age**	Elderly > middle aged > early adulthood	Decreased TST, Circadian Misalignment, SDB, Delirium
**Gender**	Female	Increased Limb Movements, Difficulty falling/staying asleep, depressed mood/anxiety
	Male	Dream Enactment Behavior, SDB, Hypersomnia, EDS
**Medical Conditions**		
*Cardiac*	Heart Failure, Myocardial Infarction, CAD	Orthopnea, SDB, Nocturia, EDS
*Gastrointestinal*	Nocturnal GER, Liver Failure	Coughing, Decreased TST, EDS, Nausea, Encephalopathy
*Endocrine*	DM, Thyroid disease	Decreased TST, Complaints of Limb Discomfort, Hypersomnolence
*Infectious*	Sepsis	Circadian Misalignment, Hypersomnolence
*Neurologic*	Dementia, Encephalitis, Epilepsy, Parkinson’s, Stroke, Demyelinating Disease	Hypersomnolence, Sundowning, Circadian Misalignment, Loss of Sleep Wake Cycles, SDB, Sleep Fragmentation
*Oncologic*	Various	Circadian misalignment, Decreased TST, Fatigue, EDS
*Pain/Palliative*	Acute and Chronic Pain	Sleep Fragmentation, Decreased TST, Medication Induced SDB
*Pulmonary*	COPD, Asthma	Early Morning Obstruction, Nocturnal Exacerbations and Desaturations, SDB
*Renal*	End Stage Renal Disease	Increased Leg Movements, Pruritis, Nausea
**Medications**		
*Cardiac*	Lipophilic beta-blockers, CNS agents, Ca++ Channel blockers, Alpha 2 Receptor Agonist, Alpha 1 Receptor Blockers, Diuretics	Nightmares, Sedation, Increased Nocturia, Decreased TST
*Neurologic*	AEDs, Anti-Parkinsons, BzRAs,	Sedating, Nightmares
*Pain*	Opiods, NSAIDs	Sedating, Enhance Obstructive and Central apneas
*Psychiatric*	Mood Stabilizers, SNRIs, SSRIs, Stimulants, TCAs	Activating, Sedating, Increase or Decrease TST
*Other*	Methlyxanthine, Antihistamines, Corticosteriods, H2 blockers, Quinolones	Activating, Sedating
**Pre-Existing Sleep Disorder**	CRD, Insomnia, OSA, PLMD, RLS	Circadian Misalignment, Decreased TST, Snoring, Apneas, Increased Arm and Leg Movements, Complaints of Limb Discomfort
**Severity of Illness**	ICU > Medical/Surgical Floor	Decreased TST, Sleep Fragmentation, Circadian Misalignment, SDB

Abbreviations: AEDs—anti-epileptic drugs, BzRAs—benzodiazepine receptor agonists, CAD—coronary artery disease, CRD—circadian rhythm disorder, DM, Diabetes Mellitus, EDS—excessive daytime sleepiness, GER—gastrointestinal reflux, ICU—intensive care unit, NSAIDs—non-steroidal anti-inflammatory drug, OSA—obstructive sleep apnea, PLMD—periodic limb movement disorder, RLS—restless leg syndrome, SDB—sleep disordered breathing, SNRI—selective serotonin norepinephrine reuptake inhibitor, SSRI—selective serotonin reuptake inhibitor, TCAs—tricyclic antidepressant, TST—total sleep time.

**Table 2:** Sleep Disorder Screening Tool [78,79,80,81,82,83].

Screening Tool	Purpose
***Adults***	
Sleep Disorder Symptom—25 Checklist (SDS-25-CL)	Comprehensive screening tool for insomnia, CRD, RLS, OSA, narcolepsy and parasomnia.
Consensus Sleep Diary	Evaluates patient reported sleep patterns
STOP-BANG	Sleep apnea screening tool
Berlin Questionnaire	Sleep apnea screening tool
Epworth Sleepiness Scale (ESS)	Excessive daytime sleepiness screening tool
Pittsburgh Sleep Quality Index (PSQI)	Evaluates subjective sleep quality and sleep habits during the last month
Functional Outcomes of Sleep Questionnaire (FOSQ)	Evaluates impact of EDS on daily activities and quality of life
Insomnia Severity Index	Evaluates severity and impact of insomnia
International Restless Legs Syndrome Scale	Evaluates severity of RLS related symptoms and their impact on sleep quality, daily affairs, and mood
***Pediatrics***	
Childhood Sleep Habits Questionnaire (CSHQ)	Parent reported evaluation of child’s sleep habits and difficulties with sleep
BEARS	B = bedtime problems, E = excessive daytime sleepiness, A = awakenings during the night, R = regularity and duration of sleep, S = snoring
ESS-CHAD (child/adolescent)	Pediatric version of ESS, evaluating daytime sleepiness

Abbreviations: CRD—circadian rhythm disorder, RLS—restless leg syndrome, OSA—obstructive sleep apnea, STOP-BANG—snoring, tired, observed apnea, high blood pressure, body mass index, age > 50, Neck size large, gender is male.

**Table 3:** Inpatient Sleep Protocol Example.

**Wake Promoting Strategies (8 am to 8 pm)**
Encourage rooms remain brightly lit
Medications scheduled for daytime dosing
Encourage out of bed and ambulation, if possible
**Sleep Preparing Strategies (8pm to 10 pm)**
Dim lighting encouraged
Encourage reduced noise levels
Encourage reduced stimulation (i.e televisions off, reduce visitation)
Medications administered, if needed
Sedating medications may be selectively administered at this time
**Sleep Protective Strategies employed between 10 pm to 4 am**
Avoid medication administration during these hours
No phlebotomy during these hours, unless urgent or necessary for obtaining trough
Increase time interval for obtaining vitals, if safe to delay (I.e q2 hr to q4hr)
Maintain hourly visual inspection schedule by nursing
**4 am to 8 am sleep is encouraged, but if medical services needed this takes precedence**
